# Preparation of feed with metal oxide nanoparticles for nanomaterial dietary exposure to fish and use in OECD TG 305

**DOI:** 10.1016/j.mex.2021.101413

**Published:** 2021-06-11

**Authors:** Azucena Bermejo-Nogales, Isabel Rucandio, Mona Connolly, María Luisa Fernández-Cruz, José María Navas

**Affiliations:** a Departamento de Medio Ambiente, Instituto Nacional de Investigación y Tecnología Agraria y Alimentaria (INIA), Carretera de la Coruña, Km 7.5, E-28040 Madrid, Spain; b Medioambientales y Tecnológicas (CIEMAT), Centro de Investigaciones Energéticas, Avenida. Complutense 22, E-28040 Madrid, Spain

**Keywords:** Bioaccumulation, Fish, Dietary exposure, Nanomaterial, Oil dispersion

## Abstract

The first step of nanomaterial accumulation in the aquatic environment is the uptake of particulate material. For substances with very low water solubility, exposure via water may be of limited relevance in comparison to the dietary route. The OECD Test Guideline 305 for bioaccumulation testing in fish using dietary exposure recommends to add substances to fish food following methodologies normally used in aquaculture (e.g. with a corn or fish oil vehicle). The feasibility of using such an approach for the testing of manufactured nanomaterials (MNs), due to their unique physical characteristics and solubility, needs to be investigated. In this study an easy, cost-effective method to prepare metal oxide nanoparticle (NP) spiked feed to give the required dietary exposure concentration to fish is described. Metal oxide NP (CeO_2_,TiO_2_ and ZnO) dispersions were prepared in oil (sunflower or olive oil) and used to soak fish feed pellets. NP surface deposition and homogeneity of distribution were analysed and confirmed. Discrepancies between nominal and measured concentrations highlighted the need to measure the achieved concentration in MN-spiked feed. The present method provides stable concentrations for bioaccumulation testing of MNs in fish through the dietary route.

A method for

• Fish feed preparation using nanomaterial-oil suspensions.
• Homogenous spiking of nanomaterials on feed.
• Nanomaterials stably maintained on feed immersed in water until eaten by fish.

## Graphical abstract

**Figure:**
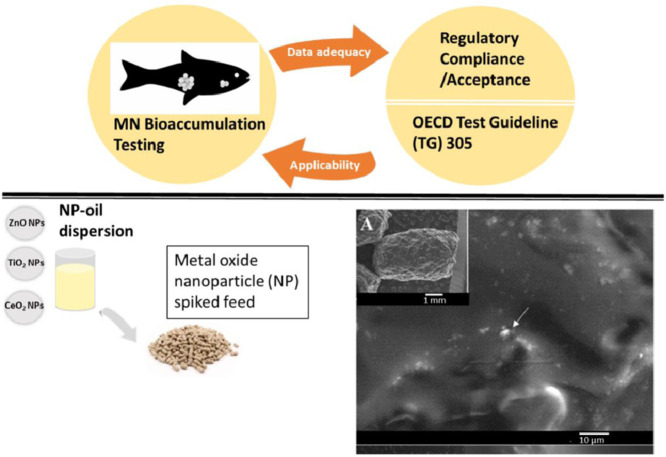

Specifications table

**Table:** 

Subject Area:	Environmental ScienceEnvironmental Science
More specific subject area:	Aquatic compartments, anthropogenic pollution, nanomaterial science, nanomaterial environmental fate and exposure
Method name:	*Nanomaterial-oil suspension feed spiking*
Name and reference of original method:	*Guidance Document on Aspects of OECD TG 305 on Fish Bioaccumulation,* OECD Environment, Health and Safety Publications, *Series on Testing & Assessment No. 264, ENV/JM/MONO(2017)16, 19-Jul-2017 [4.2.2.2 Spiking with enriched oil]*
Resource availability:	n/a

## Method details

Here we describe an easy, cost-effective method to prepare metal oxide nanoparticle (NP) spiked fish feed using an oil dispersion and soaking technique for use in manufactured nanomaterials (MN) bioaccumulation testing using the dietary exposure route. This method was developed to address an essential methodological question that remains unanswered regarding the best way to incorporate MNs into feed. An oil dispersion spiking methodology is normally used to add materials, nutrients or even chemicals to fish feed, however its use for MN spiking has been scarcely explored. Spiking of low soluble chemicals by means of corn or fish oil should be considered according to the Organization for Economic Cooperation and Development (OECD) test guideline (TG) 305 for testing bioaccumulation in fish (Section 4.2.2.2) and can also serve to adjust the dietary lipid concentration [1]. The protocols presented within this TG and its guidance document No. 264 [2] were developed for studies of dietary exposure with metals and organic chemicals, however additional considerations for MN testing have to be made. Thus, the present work concentrates on the use of oil (sunflower or olive oil) as a dispersant for MNs to allow an appropriate addition to feed pellets while avoiding any loss of MNs to the water when fish are being fed. We have focused on the characterization and appropriateness of the use of oil as a dispersant for MNs, giving enough information to potential users so that they can apply a very straightforward appropriate methodology to allow MNs to be administered to fish through the diet, for instance in the framework of the OECD TG 305 for determining bioaccumulation of MNs in fish.

## Preparation

### Nanomaterial-oil suspension preparation and characterization

Uncoated CeO_2_ NPs ((5–10 nm, 99.72% purity, 85–170 m^2^/g BET SSA) (Tecnan (Los Arcos, Spain)), TiO_2_ NPs ((NM-105) (78% anatase, 14% rutile and 8% amorphous phase) (21 nm, 99.5% purity, 50 ± 15 m^2^/g) (JRC, Ispra, Italy)) and ZnO NPs ((20–30 nm) (Tecnan, Spain)) supplied in powder form were used to prepare the NP-oil suspensions and applied to fish feed according to the developed methodology. The sunflower and olive oil were obtained in a local market, both were of the brand Eroski (Vizcaya, Spain) and described as a refined sunflower oil (0.2% maximum acidity) and a mixture of refined and virgin olive oil (0.4% maximum acidity), respectively. Preliminary investigations were performed prior to spiking the feed with the NP-oil suspensions to determine if the use of oil as a vehicle would influence NP state (e.g. aggregation state and individual particle size) compared to aqueous suspensions. Suspensions of the respective NPs prepared in Milli-Q water, sunflower oil and olive oil were characterised using a dynamic light scattering (DLS) technique (Zetasizer Nano-ZS (Malvern Instruments Ltd., UK)). Overall average hydrodynamic size distribution (Z-Ave) values, polydispersity indices and size distributions presented in intensity, number and volume (Table 1) show that oil suspensions are characterised by a monodisperse particle size distribution compared to aqueous suspensions that have polydisperse size distribution with three relative maxima.

**Table 1:** Size distribution of CeO_2_ NPs, TiO_2_ NPs and ZnO NPs in water, sunflower oil and olive oil dispersions (20 mg/mL). Polydispersity index (PdI) and Z-average values (Z-Ave) represent the width and average size of NP size distributions. Size distributions are presented according to intensity, number and volume. Number in brackets represents percentage corresponding to the peak with respect to the total signal registered. Each value is the mean ± *S*.E.M (*n* = 3).

Nanoparticles	Suspension liquid	Z-Ave (d.nm)	PdI	Average hydrodynamic diameter (d. nm ± *S*.E.M.) (%)		
				Intensity	Number	Volume
CeO_2—_NP	Water	709.8 ± 36	1	177 ± 20 (37) 1151 ± 169 (51) 5405 ± 61 (12)	137 ± 9 (96) 965 ± 160 (3) 5392 ± 100 (1)	158 ± 12 (2) 1140 ± 190 (38) 5453 ± 69 (60)
	Sunflower	629.0 ± 46	1	538 ± 43 (100)	529 ± 43 (100)	573± 50 (100)
	Olive	679.6 ± 187	1	438 ± 68 (100)	454± 91 (100)	481 ± 96 (100)
TiO_2—_NP	Water	617.4 ± 12	0.9	272 ± 25 (34) 1876 ± 514 (49) 4982 ± 168 (17)	130 ± 16 (96) 1680 ± 429(3) 4905 ± 199 (1)	134 ± 24 (1) 892 ± 333 (15) 4837 ± 406 (84)
	Sunflower	773.9 ± 4	0.5	792 ± 38 (100)	790 ± 41(100)	882 ± 39 (100)
	Olive	1134.5 ± 189	1	928 ± 132 (100)	926 ± 135 (100)	1039 ± 144 (100)
ZnO—NP	Water	2271.2 ± 173	0.64	167 ± 28 (5) 980 ± 472 (14) 4049 ± 315(81)	212 ± 60 (85) 1038 ± 546(12) 3686 ± 440 (3)	236 ± 73 (1) 592 ± 224 (1) 4345 ± 117 (98)
	Sunflower	947.1 ± 12	1	823 ± 71 (100)	800 ± 69 (100)	858 ± 73 (100)
	Olive	518.5 ± 108	1	409 ± 75 (100)	399 ± 74 (100)	422 ± 79 (100)

**Fig. 1:**
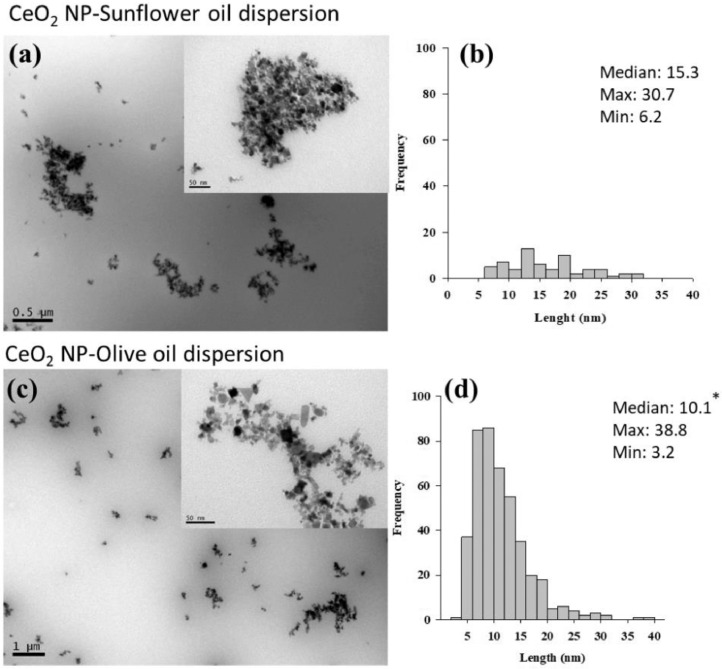
TEM analysis of CeO_2_ NP suspensions prepared in sunflower oil (20 mg/mL) (a) and olive oil (20 mg/mL) (c). Histogram showing the size frequency distribution of individual particles (b) (d) in respective oil suspensions are also presented. * indicate significant differences among particle sizes in sunflower oil or olive oil suspension (Mann-Whitney Rank Sum Test, *P*<0.05).

**Fig. 2:**
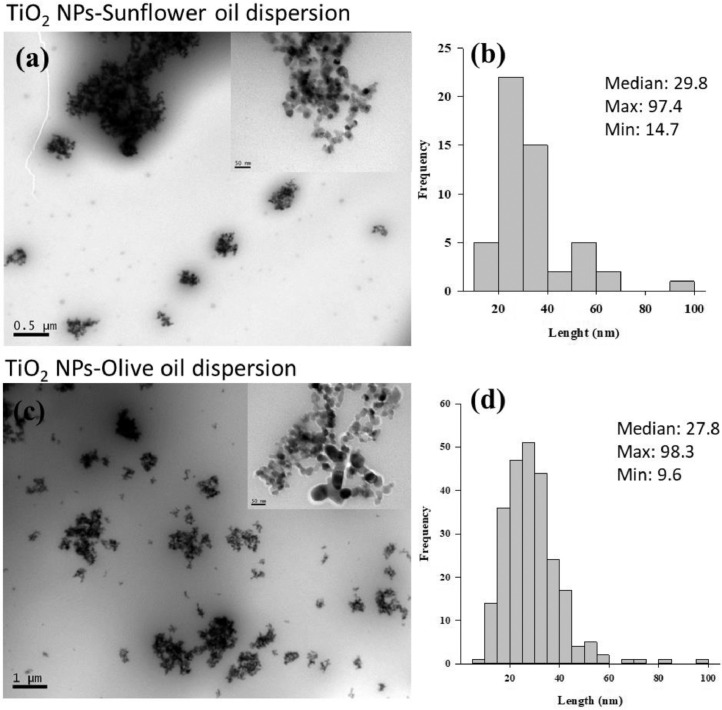
TEM analysis of TiO_2_ NP suspensions prepared in sunflower oil (20 mg/mL) (a) and olive oil (20 mg/mL)(c). Histogram showing the size frequency distribution of individual particles (b) (d) in respective oil suspensions are also presented. * indicate significant differences among particle sizes in sunflower oil or olive oil suspension (Mann-Whitney Rank Sum Test, *P*<0.05).

**Fig. 3:**
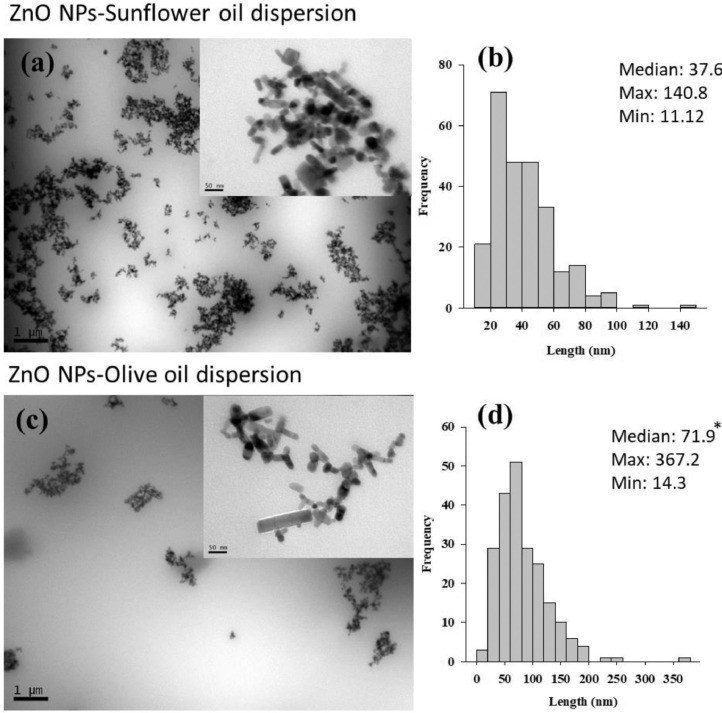
TEM analysis of ZnO NP suspensions prepared in sunflower oil (20 mg/mL) (a) and olive oil (20 mg/mL) (c). Histogram showing the size frequency distribution of individual particles (b) (d) in respective oil suspensions are also presented. * indicate significant differences among particle sizes in sunflower oil or olive oil suspension (Mann-Whitney Rank Sum Test, *P*<0.05).

Transmission electron microscopy (TEM) (JEOL 1010 HT (JEOL Ltd., Japan)) was also used to directly visualise the NP-oil suspensions following drop-coating on carbon coated copper grids (Figs. 1–3). The sizes of individual particles (200 per preparation) were measured and histograms are presented alongside the TEM images. Overall similar individual particle size distributions were measured to the pristine particle sizes reported by suppliers.

## Method details

### Procedure

*Note: This procedure uses concentrations for preparation of 60* g *of a 1000* mgNP/kg *feed test diet. While the ratio of oil to feed must be maintained to ensure homogenous coverage the stock NP concentration and quantity of feed can be adjusted according to the test diet concentration required.*

1. Combine 60 mg of nanomaterial (powder) in 3 mL of sunflower or olive oil in a 15 mL conical centrifuge tube. Vortex at 2500 rpm for 1 min to ensure homogenous dispersion. The resulting concentration is 20 mg NP/mL (2% w/v).
2. Weigh 60 g of fish feed (pellets, 3 mm) and place in a 250 mL glass beaker.
3. Pour the NP-oil suspension over the feed [spike volume to feed ratio of 50 uL per gram feed] and mix using a glass rod.
4. The suspension will be viscous and may require some time to drain out. There will be a quantity left in the tube that cannot be avoided (as evidenced in this study for CeO_2_, TiO_2_, ZnO NPs).
5. Mix using a glass rod to facilitate even distribution and complete coverage of the pellets with the NP-oil suspension.
6. Leave soaked pellets to dry (room temperature overnight).
7. Store pellets in an airtight container (protected from light) at 4 °C once prepared for use and throughout the bioaccumulation study (stability can be checked by analysing pellets at various time points throughout the study).

## Method validation

The method developed was applied using CeO_2_, TiO_2_ and ZnO NP powders and the concentration of respective metal in the spiked feed was analysed to assess overall recovery and homogeneity of coverage. As well as overall recovery in spiked feed any deviations from nominal concentrations prepared in NP-oil suspensions and any potential losses to labware was assessed by measuring concentrations in NP-oil suspensions before use and any NP residues in glassware washed with acetone. Inductively coupled plasma atomic emission spectroscopy (ICP-OES) analysis (Agilent 5100 model (Agilent Technologies Spain, S.L., Spain)) was used to measure the concentrations. In brief, feed samples were acid digested using a mixture of nitric acid (HNO_3_, 65% w/w), hydrofluoric acid (HF, 48% w/w) and hydrogen peroxide (H_2_O_2_, 30% w/w) at a proportion of 77.7%:11.1%:11.1%, respectively. Digestions were performed in a DigiPrep system using a temperature ramp cycle first to maintain 75 °C for 15 min, followed by dropwise addition of the H_2_O_2_ and thereafter an increase in temperature to 115 °C and maintained for 60 min. NP-oil sock suspensions and any NP residues impregnated in the containers (washed with acetone) were also acid digested (71.4% (HNO_3_, 65% w/w): 14.28% ((HF, 48% w/w): 14.28% (NP-oil suspension or 1.42% NP-oil residues)) in HVT50 vessels using a rotor 12HVT50 in a Microwave Digestion System Multiwave 60 (Anton Paar Spain S.L.U., Spain). The amounts of Ce, Ti, and Zn in the initial NP-oil suspensions as well as leftover in the labware used (tubes and beakers) are presented in Table 2. A good agreement between nominal and measured concentrations for the TiO_2_ and ZnO NP-oil suspensions was seen. The increase in concentrations measured above nominal concentrations is due to the background levels of Ti already present in the sunflower and oil vehicles (8.50 and 16.38 mg/L respectively). However, a loss of 58% and 44% from the nominal concentration in NP sunflower and olive oil suspensions, respectively was measured in CeO_2_ NP-oil stocks. This discrepancy could be related to an unstable suspension, difficulty in digestion or loss during measurement (e.g. interaction with equipment tubing etc.). Also some losses (≤ 9.7%) during the preparation of the spiked feed, to tubes and beakers were evidenced. This highlights the necessity to measure the actual concentration in spiked pellets in order not to overlook potential losses/discrepancies often seen when working with unstable NPs.

**Table 2:** Amounts of Ce, Ti or Zn in NP oil stock dispersions and left in labware (tubes and beakers) used to spike feed pellets. Values are mean ± *S*.E.M of three samples.

Amount of metal (mg total)		Sunflower oil		Olive oil	
	Nominal	Measured	% loss	Measured	% loss
**Cerium**					
Stock	48.85	20.50 ± 0.32	58	27.50 ± 1.77	44
Empty tube		1.72 ± 0.04	3.5	1.61 ± 0.35	3.3
Empty beaker		0.13 ± 0.01	0.3	0.14 ± 0.03	0.3
**Titanium**					
Stock	35.96	39.98 ± 1.23	11 (+)	38.80 ± 1.88	7.9 (+)
Empty tube		2.40 ± 0.33	6.7	3.49 ± 0.54	9.7
Empty beaker		1.08 ± 0.24	3	1.79 ± 0.36	4.9
**Zinc**					
Stock	48.20	45.89 ± 2.52	4.8	50.66 ± 1.23	5.1 (+)
Empty tube		4.60 ± 0.42	9.5	3.13 ± 0.36	6.5
Empty beaker		1.56 ± 0.16	3.2	0.82 ± 0.14	1.7

## Stability of spiking

Following application of the developed methodology leaching experiments were performed with the spiked feed pellets to determine the extent to which they become stably coated and if any potential losses to the water column occur if applied in testing. The concentrations of metal in NP spiked feed achieved following spiking (pellet time 0 min), following immersion in aquarium water for a short duration (5 min) and longer duration (24 h) was measured and expressed as either% recovery of the expected concentration in feed or as% loss of the actual measured concentration in spiked pellets (Table 3). Expected concentrations in pellets before immersion were calculated taking into consideration the concentrations lost in labware using sunflower and olive oil as dispersants, respectively. Using sunflower oil as a dispersant recovery varied from almost complete recovery for CeO_2_ and ZnO NP spiked feed (93.5 and 96.8%, respectively) to a poor recovery (60–75%) in the case of TiO_2_ NP spiking's. Poor recovery (67.9–76.7%) was seen in all cases when using olive oil as a vehicle. Pellets measured after 24 h immersion maintained the same recovery ± 9.7% as spiked pellets at time 0 min. These values also represented ≤15% losses from measured concentrations, after 24 h immersion.

**Table 3:** Concentrations of Ce, Ti or Zn in spiked pellets measured following spiking feed pellets, after 5 min and 24 h soaking (leaching experiment). Values are mean ± *S*.E.M of three samples.

Concentration of metal (mg/g)		Sunflower oil			Olive oil			
	Expected*	Measured	% recovery^	% loss#	Expected*	Measured	% recovery^	% loss#
**Cerium**								
Pellet time 0 min	0.31	0.29 ± 0.01	93.5		0.43	0.33 ± 0.06	76.7	
Pellet time 5 min		0. 27 ± 0.04	87.1	6.9		0.28 ± 0.04	65.1 15.2	
Pellet time 24 h		0.27 ± 0.04	87.1	6.9		0.29 ± 0.04	67.4 12.1	
**Titanium**								
Pellet time 0 min	0.61	0.37 ± 0.07	60.6		0.56	0.42 ± 0.03	75.0	
Pellet time 5 min		0.40 ± 0.02	65.6	8.0 (+)		0.41 ± 0.04	73.2 2.4	
Pellet time 24 h		0.36 ± 0.03	59.0	2.7		0.45 ± 0.07	80.3 7.1	
**Zinc**								
Pellet time 0 min	0.62	0.60 ± 0.07	96.8		0.78	0.53 ± 0.04	67.9	
Pellet time 5 min		0.50 ± 0.03	80.6	16.7		0.49 ± 0.04	62.8 7.6	
Pellet time 24 h		0.54 ± 0.01	87.1	10.0		0.55 ± 0.03	70.5 3.8(+)	

⁎ Concentration of metal in the pellet according to measured content in stock and subtracting losses to labware (e.g. stock amount – (tube+ beaker amounts)), ^in relation to expected concentration.

# from measured concentration

## Detection of NPs in spiked feed pellets

**Fig. 4:**
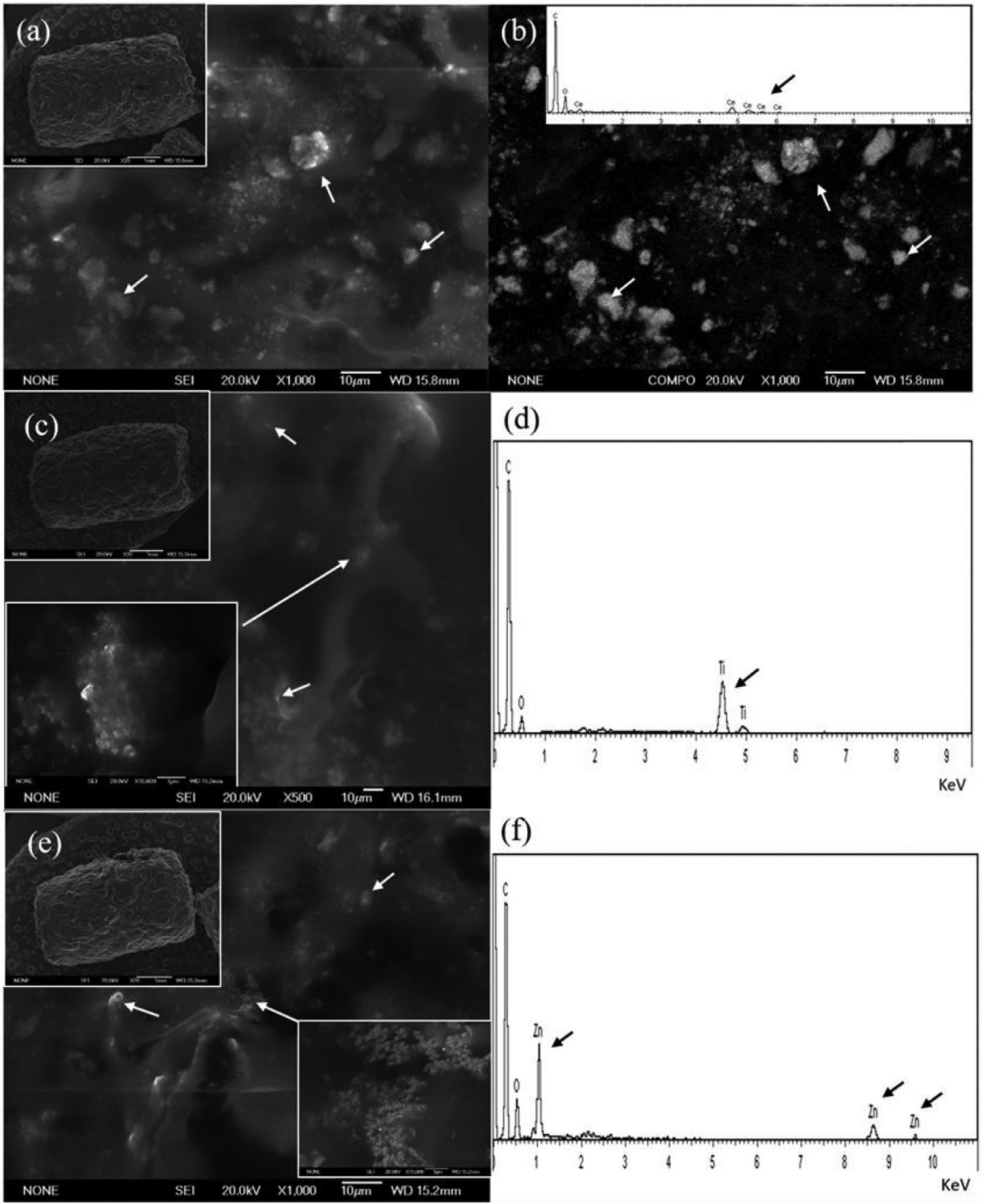
SEM micrographs of NP-olive oil dispersion soaked pellets along with respective EDX elemental analysis. Micrographs of CeO_2_ NP soaked pellets (scale bars 1 mm and 10 µm) (a), and respective EDX and BSE analysis showing the presence of cerium on pellets (b). TiO_2_ NP soaked pellets (scale bars 1 mm, 1 µm and 10 µm) (c) and EDX analysis showing the presence of titanium on pellets (d). ZnO NP soaked pellets (scale bars 1 mm, 1 µm and 10 µm) (e) and EDX analysis showing the presence of zinc on pellets (f).

Following preparation, feed pellets were also analysed to detect the presence of the respective NPs (CeO_2_, TiO_2_, and ZnO NPs) on the surface using scanning electron microscopy (SEM). Fig. 4 shows results obtained with NP-olive oil suspensions. Similar results were obtained with sunflower oil and are presented in the supplementary material (Figure S1). In all cases, pellets were homogeneously covered by these NPs, as it was not possible to detect regions not covered or with a greater accumulation of NPs. The presence of Ce, Ti or Zn was confirmed by means of energy dispersive x-ray micro-analysis (EDX). In addition, in the case of CeO_2_ NPs, aggregates adsorbed to the pellet were evidenced by means of back-scattered electrons (BSE) (Fig. 4B).

## Additional information

The here presented developed methodology can be used to disperse NPs (e.g. metal oxide MNs) in oil suspensions (sunflower or olive oil) with minimal manipulation (vortexing) and following a simple mixing step NPs are stably incorporated onto the spiked feed pellet surface. An additional advantage of this technique is that the NP form is maintained and the oil itself can act as a coating preventing leaching. It provides much needed methodological guidance to spike feed when testing the bioaccumulation of low solubility, difficult to handle materials such as MNs. However, care must be taken regarding specific particle types and properties that may influence spiking recovery (e.g. size) and thus following application of the spiking methodology, the recovered concentration must be measured. Overall this method for NP feed spiking contributes to developing NP specific reference for inclusion in a guidance document related to the testing of bioaccumulation potential of MNs in fish through the dietary route. Other methodologies that have been explored include spikings with preparations in aqueous dispersion with or without the use of chemical dispersants or dispersing aids (e.g. sonication), and also the direct incorporation of NMs in powder form, along with other feed constituents, in a paste followed by extrusion. In cases where aqueous dispersion spiking's were used, an additional top coating step (e.g. gelatine coating) may be needed to prevent leaching from the feed. The direct powder addition approach allows the customisation of food pellets but it is also more labour-intensive.

## Appendix Supplementary materials

Image, application 1

## References

[1] OECD (2017). Guidance Document on Aspects of OECD TG 305 on Fish Bioaccumulation. http://www.oecd.org/officialdocuments/publicdisplaydocumentpdf/?cote=ENV/JM/MONO(2017)16&doclanguage=en.

[2] OECD, Test No. 305: Bioaccumulation in Fish: Aqueous and Dietary Exposure, OECD Guidelines for the Testing of Chemicals, Section 3 a, OECD Publishing, Paris, 2012 [cited Nov 23, 2020] Available from: doi:10.1787/9789264185296-en.

