# Supplementary material for: Preparation of feed with metal oxide nanoparticles for nanomaterial dietary exposure to fish and use in OECD TG 305
Source: MethodsX. 2021 Jun 11;8:101413. doi: 10.1016/j.mex.2021.101413 (PMC8374479; doi:10.1016/j.mex.2021.101413)
Supplement: Supplementary file 1 [file mmc1.docx]

**Supplementary material**

**
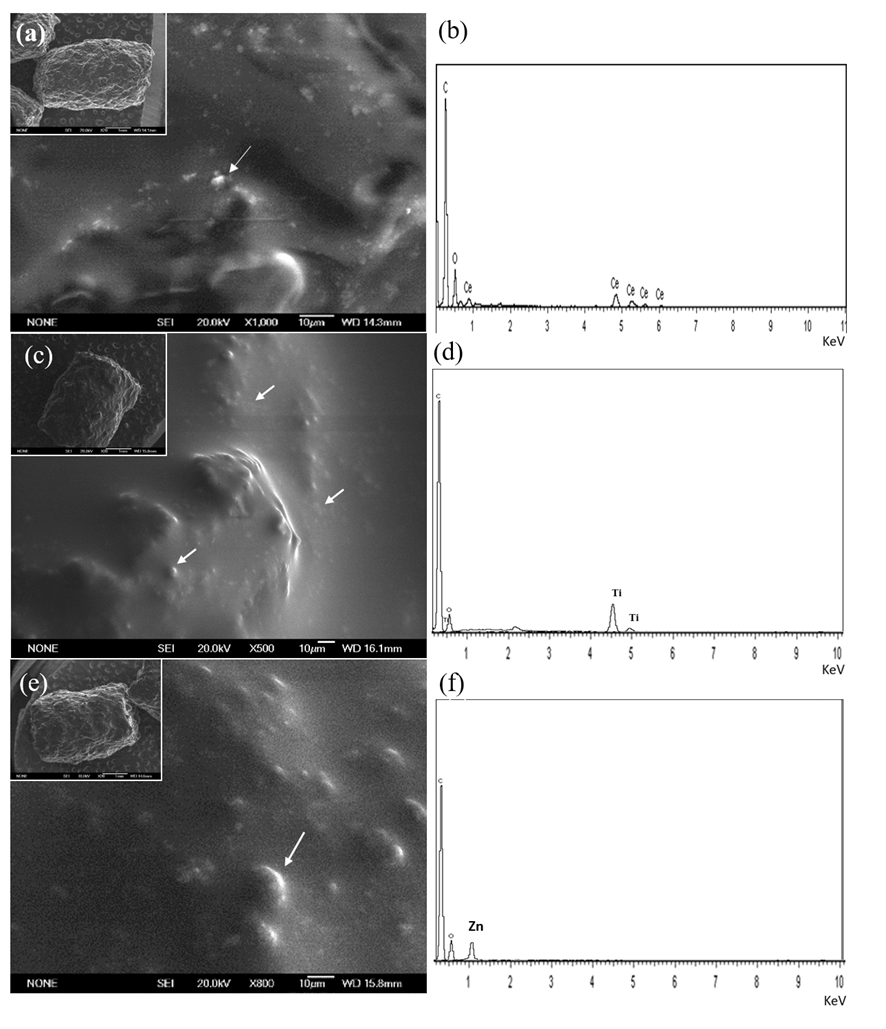
**

**Figure S1.** SEM micrographs of NP-sunflower oil dispersion soaked pellets along with respective EDX elemental analysis. Micrographs of CeO_2_ NP soaked pellets (scale bars 1 mm and 10 µm) (a), and respective EDX analysis showing the presence of cerium on pellets (b). TiO_2_ NP soaked pellets (scale bars 1 mm and 10 µm) (c) and EDX analysis showing the presence of titanium on pellets (d). ZnO NP soaked pellets (scale bars 1 mm and 10 µm) (e) and EDX analysis showing the presence of zinc on pellets (f).
